# A Pharmacogenetics-Based Warfarin Maintenance Dosing Algorithm from Northern Chinese Patients

**DOI:** 10.1371/journal.pone.0105250

**Published:** 2014-08-15

**Authors:** Jinxing Chen, Liying Shao, Ling Gong, Fang Luo, Jin'e Wang, Yi Shi, Yu Tan, Qianlong Chen, Yu Zhang, Rutai Hui, Yibo Wang

**Affiliations:** 1 State Key Laboratory of Cardiovascular Disease, Sino-German Laboratory for Molecular Medicine, Fuwai Hospital, National Center for Cardiovascular Diseases, Chinese Academy of Medical Sciences and Peking Union Medical College, Beijing, China; 2 Department of Cardiology, Fuwai Hospital, National Center for Cardiovascular Diseases, Chinese Academy of Medical Sciences and Peking Union Medical College, Beijing, China; 3 Department of Cardiovascular Surgery, Fuwai Hospital, National Center for Cardiovascular Diseases, Chinese Academy of Medical Sciences and Peking Union Medical College, Beijing, China; University of Florida, United States of America

## Abstract

Inconsistent associations with warfarin dose were observed in genetic variants except *VKORC1* haplotype and CYP2C9*3 in Chinese people, and few studies on warfarin dose algorithm was performed in a large Chinese Han population lived in Northern China. Of 787 consenting patients with heart-valve replacements who were receiving long-term warfarin maintenance therapy, 20 related Single nucleotide polymorphisms were genotyped. Only *VKORC1* and *CYP2C9* SNPs were observed to be significantly associated with warfarin dose. In the derivation cohort (n = 551), warfarin dose variability was influenced, in decreasing order, by *VKORC1* rs7294 (27.3%), CYP2C9*3(7.0%), body surface area(4.2%), age(2.7%), target INR(1.4%), *CYP4F2* rs2108622 (0.7%), amiodarone use(0.6%), diabetes mellitus(0.6%), and digoxin use(0.5%), which account for 45.1% of the warfarin dose variability. In the validation cohort (n = 236), the actual maintenance dose was significantly correlated with predicted dose (r = 0.609, *P*<0.001). Our algorithm could improve the personalized management of warfarin use in Northern Chinese patients.

## Introduction

Warfarin has remained the mainstay of oral anticoagulant therapy for the treatment and prevention of thromboembolism. However, management of warfarin therapy is challenging because of its narrow therapeutic index and wide inter-individual variability. The correct dosing is necessary to avoid bleeding or risk of thrombotic events in case of an excessive or a too low dose. Many studies have shown that single nucleotide polymorphisms (SNPs) within *CYP2C9* (cytochrome P450, family 2, subfamily C, polypeptide 9) and *VKORC1* (vitamin K epoxide reductase complex, subunit 1) genes are related to warfarin dose requirement [1], [2], [3], [4]. These two genes in combination with age, gender, and body mass index have been shown to account for 30–50% of the variability in the dosage of warfarin and acenocoumarol [1], [4], [5], [6], [7], [8], [9], [10]. In addition to *VKORC1* and *CYP2C9* SNPs, *CYP4F2* (cytochrome P450, family 4, subfamily F, polypeptide 2) rs2108622(V433M) was found to be associated with warfarin dose in 3 independent Caucasian cohorts and accounted for the difference in warfarin dose of approximately 1 mg/day between CC and TT subjects [11]. Several genome-wide association studies indicated that there seemed to be no common SNPs with large effects on warfarin maintenance dose outside of the *CYP2C9*, *VKORC1*, and *CYP4F2* [12], [13], [14], [15]. A recent association study with a Chinese population also confirmed this observation [16]. Candidate gene studies have also detected associations between other SNPs and warfarin dose, such as SNPs in *CYP2C19* (cytochrome P450, family 2, subfamily C, polypeptide 19) [17], *GGCX* (gamma-glutamyl carboxylase) [18], [19], [20], [21], *EPHX1* (epoxide hydrolase 1) [21], [22], [23], [24], *CALU* (calumenin) [25], and *MPZ* (myelin protein zero) [12]. However, the associations of these SNPs with warfarin dose were inconsistent. In *CYP4F2* gene, a functional haplotype block represented by rs3093105 was found to be associated with urinary 20-hydroxyeicosatetraenoic acid (20-HETE) and hypertension in Chinese individuals [26], while rs2074902 in the haplotype block and rs3093158 were found to be associated with Crohn's disease [27]. We also included some functional SNPs in related genes including *F2* (coagulation factor II), *F5* (coagulation factor V), *F11* (coagulation factor XI), and *PROCR* (protein C receptor), whose proteins are involved in thrombosis. Previous studies constructed many algorithms to predict the warfarin maintenance dose in Chinese individuals [16], [28], [29], [30], [31], [32]. However, they frequently used relatively small populations (<400 subjects) except in two studies where one included 845 from Southern China [16], and the other included 641 from Central China [33]. The two algorithms included different factors and could explain the 43.6% [16] and 56.4% [33] variability in warfarin maintenance dose, respectively. The objective of the current study was to assess these genetic determinants of the warfarin maintenance dose and to construct an algorithm integrating common interference factors to predict the dose in a large population who lived in Northern China.

## Materials and Methods

### Ethics Statement

All subjects provided their written informed consent. The study protocol was conducted in accordance with the Declaration of Helsinki Principles (revised in 1983), reviewed and approved by the Ethics Committee of Fuwai Hospital, National Center for Cardiovascular Diseases, Chinese Academy of Medical Sciences, Beijing, China.

### Study design

Consenting patients with heart valve replacements were recruited, and 20 single nucleotide polymorphisms (SNPs) in related genes were examined. First, the associations of these SNPs with the warfarin maintenance dose were tested. Second, on the basis of genotypes associated with the warfarin maintenance dose, an algorithm integrating common non-genetic factors was constructed to predict the dose in the derivation cohort (70% of the whole cohort), and was assessed in the validation cohort (30% of the whole cohort).

### Study population

Inpatients aged 18 and over, were consecutively recruited from patients undergoing heart valve replacement (HVR) therapy at Fuwai Hospital from April 2012 to May 2013. All patients received warfarin therapy for at least 3 months, and provided records for INR and the stable maintenance dose defined as a constant dose for at least 1 month with the INR measurements within the range of 1.6–2.5 for multiple time periods after hospitalization. All subjects had self-reported as Han nationality and having lived in Northern China.

We collected data on clinical factors which have previously been associated with warfarin dose through patient interviews and a review of medical records by a trained physician. These factors included age, gender, height, weight, current smoking habits and alcohol consumption, concomitant diseases and concurrent interacting medications. The body surface area (BSA) was calculated by height and weight using the following equation: BSA (m^2^) = 0.0061× height (cm) +0.0128× weight (kg) − 0.1529. The disease was defined as a diagnosis or treatment for the corresponding disease. Hypertension was diagnosed after taking a mean of 3 independent measures of blood pressure >140/90 mmHg; diabetes mellitus (DM) was diagnosed when the subject had a fasting glucose >7.0 mmol/L, or >11.1 mmol/L at 2 hours after oral glucose challenge, or both; hyperlipidemia was diagnosed with an elevation of at least one of the following: >6.22 mmol/L for total cholesterol, >2.26 mmol/L for triglycerides, or >4.14 mmol/L for LDL-cholesterol. Patients with hematological diseases, peptic ulcers, liver and kidney dysfunctions, infections, autoimmune diseases, and malignant tumors were excluded from this study.

### Genotype analysis

The RelaxGene Blood DNA System DNA isolation kit (Tiangen, Beijing, China) was used for preparing genomic DNA following the recommendations of the manufacturer. The SNP rs7294 was selected to reflect the natural haplotype block for genotyping according to our previous study [34]. Genotyping was performed using the MassARRAY high-throughput DNA analysis system with matrix-assisted laser desorption/ionization time-of-flight mass spectrometry (Sequenom, Inc., San Diego, CA, USA). The primers were designed using MassARRAY Assay Design software (version 3.1). SNPs were genotyped using iPLEX Gold technology (Sequenom) followed by automated data analysis with the TYPE RT software version 4.0. Three samples were removed due to failed genotyping.

### Statistical analyses

Categorical variables were reported as counts (percentages) and continuous variables were reported as medians (Q1 to Q3) where appropriate. The differences between the derivation and validation cohorts were calculated using the Wilcoxon rank-sum test or χ^2^-test. According to the time when they were enrolled, early enrolled patients (70%) were selected for deriving the dose algorithm for warfarin maintenance dose; the remaining later enrolled patients were selected for validating the algorithm by comparing the actual maintenance doses and predicted doses. Univariate association between the warfarin maintenance dose and each potential predictor was assessed using linear regression analysis. All SNPs were tested for deviations from Hardy-Weinberg equilibrium using the χ^2^-test, and for their association with the warfarin dose by Spearman correlation analysis using a co-dominant model. Categorical variables were coded as 1 if present and 0 if absent. The variables of the variant allele were coded as 0, 1, and 2 for zero copy, one copy, and two copies of the variant allele. Those potential predictors with a *P*-value lower than 0.20 were selected as candidate variables for the algorithm by a stepwise multiple regression method in the derivation cohort. The algorithm was validated in the validation cohort using Pearson correlation analysis. We compared the performance of the algorithm in three subsets of the entire cohort, distributed according to the warfarin dose requirement (low dose: < = 2 mg/day; intermediate dose: >2 and <4 mg/day; high dose: > = 4 mg/day). The thresholds of 2 mg and 4 mg per day cover the usual starting dose of 3 mg per day used for Chinese individuals. We evaluated the potential clinical value of our algorithm in three different dose groups by calculating the percentage of patients whose predicted warfarin dose was within 20% of the actual maintenance dose (ideal dose), at least 20% higher (overestimation) or 20% lower (underestimation) than the actual dose. A two-tailed probability value of <0.05 was considered as significant. The analyses were performed using SPSS 13.0 for Windows.

## Results

### Characteristics of enrolled patients

A total of 800 patients were initially enrolled. Of these, 10 patients were excluded because they did not achieve a stable anticoagulation in the follow-up, 3 patients were excluded because a low call rate was in their DNA genotyping. The patients' characteristics showed that 70% patients (n = 551) were enrolled in the derivation cohort and 30% patients (n = 236) were in the validation cohort (Table 1). Usually, most HVR patients with heart failure were treated with digoxin for 3–6 months after hospitalization. Digoxin therapy would be stopped if their heart function recovered. The patients in the derivation cohort were enrolled earlier, thus the ratio of patients using digoxin was much lower in the derivation cohort than that in the validation cohort.

**Table 1:** Study subjects characteristics.

Variable	Median (Q1–Q3) or number (%)		
	Derivation cohort	Validation cohort	*P* *
Number	551	236	
Dose, mg/day	3.00(2.25–3.75)	3.00(2.25–3.75)	0.281
Target INR	2.00(1.80–2.15)	1.98(1.81–2.20)	0.902
Age, yrs	51 (43–60)	54(44–60)	0.634
Male, %	308(55.9%)	114(48.3%)	0.050
Body surface area, m^2^	1.71(1.57–1.86)	1.68(1.55–1.82)	0.069
Cigarette Smoking, %	168(30.5)	70(29.7)	0.816
Alcohol drinking, %	97(17.6)	38(16.1)	0.608
Most common concurrent interacting medications			
Digoxin, %	89(16.2)	103(43.6)	<0.001
Amiodarone, %	16(2.9)	10(4.2)	0.338
Amlodipine, %	15(2.7)	6(2.5)	0.886
Diltiazem, %	11(2.0)	3(1.3)	0.481
Simvastatin, %	4(0.7)	1(0.4)	0.625
Most common comorbidities,			
Atrial fibrillation, %	219(39.7)	85(36.0)	0.325
Hypertension, %	119(21.6)	49(20.8)	0.794
Coronary artery disease, %	81(14.7)	34(14.4)	0.999
Hyperlipidemia, %	60(10.9)	21(8.9)	0.400
Diabetes, %	43(7.8)	16(6.8)	0.617
Valve position			0.329
Mitral valve, %	222(40.3)	104(44.1)	
Aortic valve, %	201(36.5)	70(29.7)	
Tricuspid valve, %	4(0.7)	2(0.8)	
Combined valve, %	124(22.5)	60(25.4)	

*The difference between the derivation and validation cohorts was calculated using the Wilcoxon rank-sum test and the χ^2^-test.

### Genetic determinants affecting the warfarin maintenance dose

Due to a nature haplotype block in the *VKORC1* gene in the Chinese population, the rs7294 SNP reflecting the haplotype was selected for genotyping. Table 2 shows the relationships between the candidate SNPs and the warfarin maintenance dose in the derivation cohort. *VKORC1* rs7294, CYP2C9*3 rs1057910, and *CYP2C9* rs4917639 were significantly associated with the warfarin maintenance dose. In related genes, such as *CYP4F2*, *GGCX*, *CALU*, *CYP2C19*, *EPHX1*, *F2*, *F5*, *F11*, and *PROCR*, we could not confirm previous associations of these SNPs with warfarin dose in our Chinese population. However, *CYP4F2* rs2108622, *EPHX1* rs4653436, and *F2* rs3136516 showed an insignificant correlation with *P*<0.20.

**Table 2:** Association of candidate SNPs with warfarin maintenance dose in the derivation cohort.

								Spearman*	
SNP	Gene	Genotype	Number (%)	MAF(%)	HWE *P*	Mean(SE)	Median	rho	*P*
rs7294	VKORC1	GG	448(81.3)	9.5	0.14	2.82(0.04)	3.00	0.424	<0.001
		AG	101(18.3)			4.33(0.15)	4.12		
		AA	2(0.4)			9.75(0.75)	9.75		
rs1057910	CYP2C9	AA	506(91.8)	4.2	0.97	3.21(0.06)	3.00	−0.261	<0.001
		AC	44(8.0)			2.13(0.14)	2.00		
		CC	1(0.2)			2.00	2.00		
rs4917639	CYP2C9	AA	466(84.6)	8.3	0.21	3.22(0.06)	3.00	−0.204	<0.001
		AC	79(14.3)			2.55(0.12)	2.25		
		CC	6(1.1)			2.52(0.44)	2.00		
rs2108622	CYP4F2	GG	288(52.3)	28.3	0.31	3.03(0.07)	3.00	0.076	0.073
		AG	214(38.8)			3.23(0.09)	3.00		
		AA	49(8.9)			3.16(0.16)	3.00		
rs4653436	EPHX1	GG	349(63.5)	20.1	0.56	3.20(0.07)	3.00	0.091	0.064
		AG	181(32.9)			2.97(0.09)	3.00		
		AA	20(3.6)			3.20(0.38)	3.00		
rs2290228	CALU	CC	334(61.2)	22.4	0.17	3.16(0.07)	3.00	−0.038	0.373
		CT	179(32.8)			3.11(0.10)	3.00		
		TT	33(6.0)			2.80(0.14)	3.00		
rs3093105	CYP4F2	TT	436(79.1)	11.2	0.62	3.10(0.06)	3.00	0.001	0.987
		GT	107(19.4)			3.18(0.15)	3.00		
		GG	8(1.5)			3.05(0.25)	3.00		
rs9332127	CYP2C9	GG	509(92.5)	3.9	0.19	3.13(0.05)	3.00	0.018	0.678
		GC	39(7.1)			3.07(0.16)	3.00		
		CC	2(0.4)			1.69(0.94)	1.69		
rs3093158	CYP4F2	AA	169(30.8)	44.9	0.69	3.20(0.10)	3.00	−0.046	0.277
		AG	267(48.6)			3.12(0.08)	3.00		
		GG	113(20.6)			3.01(0.09)	3.00		
rs4244285	CYP2C19	GG	262(47.8)	30.5	0.56	3.07(0.08)	3.00	−0.020	0.647
		AG	238(43.4)			3.19(0.09)	3.00		
		AA	48(8.8)			3.08(0.18)	3.00		
rs4986893	CYP2C19	GG	502(91.1)	4.6	0.43	3.12(0.06)	3.00	0.009	0.838
		AG	47(8.5)			3.18(0.20)	3.00		
		AA	2(0.4)			2.25(1.50)	2.25		
rs339097	CALU	TT	527(95.8)	2.1	0.62	3.14(0.06)	3.00	−0.036	0.400
		TC	23(4.2)			2.83(0.19)	3.00		
rs699664	GGCX	GG	273(49.8)	29.5	0.93	3.18(0.08)	3.00	0.054	0.210
		AG	227(41.4)			3.05(0.08)	3.00		
		AA	48(8.8)			3.11(0.25)	2.88		
rs12714145	GGCX	CC	261(47.5)	31.7	0.36	3.12(0.08)	3.00	0.010	0.810
		CT	229(41.6)			3.10(0.08)	3.00		
		TT	60(10.9)			3.18(0.20)	3.00		
rs2292566	EPHX1	GG	281(51.0)	28.5	0.88	3.15(0.08)	3.00	−0.007	0.864
		AG	226(41.0)			3.12(0.08)	3.00		
		AA	44(8.0)			2.94(0.17)	3.00		
rs3756009	F11	AA	301(54.7)	25.5	0.38	3.15(0.07)	3.00	−0.013	0.756
		AG	217(39.5)			3.05(0.08)	3.00		
		GG	32(5.8)			3.36(0.28)	3.00		
rs7542281	F5	CC	346(63.1)	20.9	0.43	3.11(0.07)	3.00	0.000	0.992
		CT	175(31.9)			3.17(0.10)	3.00		
		TT	27(4.9)			2.93(0.18)	3.00		
rs3136516	F2	GG	354(64.5)	20.1	0.32	3.18(0.07)	3.00	0.070	0.103
		AG	169(30.8)			3.01(0.10)	3.00		
		AA	26(4.7)			3.14(0.31)	3.00		
rs1415774	PROCR	AA	251(45.8)	33.1	0.26	3.18(0.08)	3.00	−0.014	0.739
		AG	231(42.2)			3.03(0.08)	3.00		
		GG	66(12.0)			3.22(0.16)	3.00		
rs12065184	MPZ	AA	429(78.1)	11.7	0.56	3.09(0.06)	3.00	0.025	0.560
		AC	111(20.2)			3.29(0.12)	3.00		
		CC	9(1.6)			2.51(0.29)	2.25		

MAF means minor allele frequency, HWE means Hardy-Weinberg equilibrium.

*All SNPs were tested for association with warfarin dose by Spearman correlation analysis using a codominant model.

**Figure 1:**
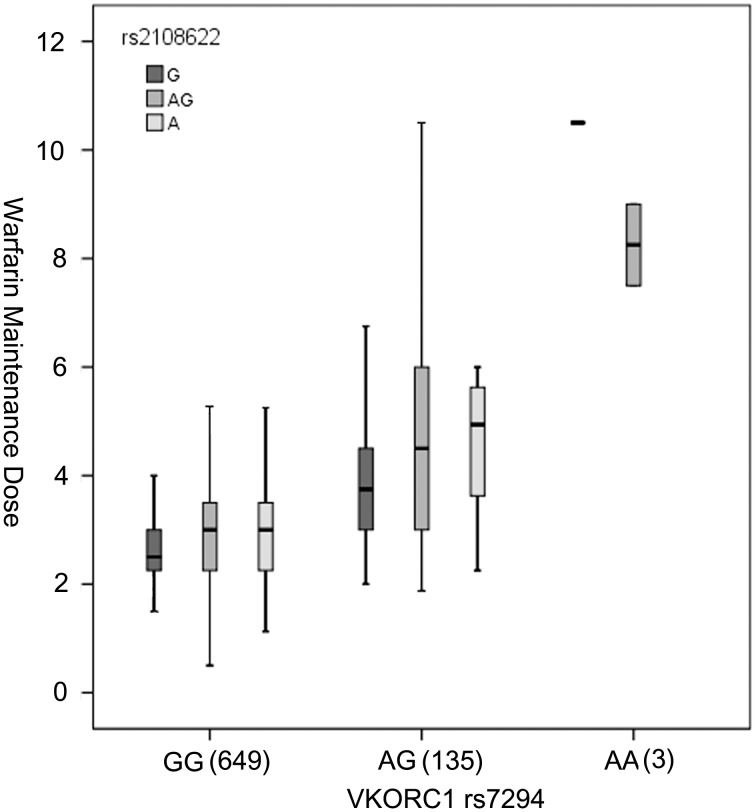
The effect of *CYP4F2* rs2108622 on warfarin maintenance dose grouped by *VKORC1* rs7294 genotypes (mg/day). Each box indicates the 25th to 75th percentile values (interquartile range); the black lines represent the median daily warfarin maintenance dose value, the maximum length of the whisker is 1.5 times the interquartile range. In detail below are the specific statistical significances for each group. *VKORC1* rs7294 GG: *P*_ANOVA_ = 0.030; *P*_Spearman_ = 0.005. *VKORC1* rs7294 AG: *P*_ANOVA_ = 0.031, *P*_Spearman_ = 0.027.

We further evaluated the effect of rs2108622 on warfarin dose in patients grouped by *VKORC1* rs7294 due to the significant effect of the *VKORC1* haplotype on warfarin dose. In patients with rs7294 wild-type genotype GG (n = 649), the results showed a significant difference (*P* for ANOVA was 0.030) and correlation (*P* for Spearman was 0.005). In patients with the rs7294 heterozygotes genotype (n = 135), the results also showed a significant difference (*P* for ANOVA was 0.031) and correlation (*P* for Spearman was 0.027) (Figure 1).

### Multiple regression model for warfarin maintenance dose

In the derivation cohort, we first analyzed the correlation between non-genetic factors and the warfarin maintenance dose. The factors with a linear regression *P*-value <0.20 including age, gender, BSA, target INR, amiodarone, digoxin, and DM are shown in Table 3. Adding 6 genetic factors (*VKORC1* rs7294, CYP2C9*3 rs1057910, *CYP2C9* rs4917639, *CYP4F2* rs2108622, *EPHX1* rs4653436, and *F2* rs3136516) with *P*-value less than 0.20, a total of 13 factors were carried in the stepwise regression analyses of the derivation cohort (n = 551). Finally, 9 factors included in the regression model were listed in Table 4. In this model, the *VKORC1* and *CYP2C9* genetic factors contributed most (27.3% and 7%, respectively) to the inter-individual variability in warfarin dose. Age and BSA, of the non-genetic factors, contribute most (4.2% and 2.7%, respectively) to the inter-individual variability. To derive a patient's dose using our model, a clinician would complete the following algorithm using the patient's clinical and genetic characteristics:

**Table 3:** Non-genetic factors influencing the warfarin maintenance dose.

Factors	Coefficient*	*P*
Age	−0.195	<0.001
BSA	0.159	<0.001
Sex	−0.073	0.085
Amiodarone use	−0.104	0.015
Digoxin use	−0.086	0.044
Diabetes mellitus	0.069	0.104
Target INR	0.065	0.125

*The correlation was analyzed with linear regression.

**Table 4:** Final model produced by stepwise regression analysis.

Variable	Partial R^2^	*P*	Coefficient B (95%CI)
*VKORC1* rs7294	0.273	<0.001	1.787(1.584, 1.990)
*CYP2C9* rs1057910	0.070	<0.001	−1.213(−1.500, −0.925)
Body surface area	0.042	<0.001	1.315(0.896, 1.734)
Age	0.027	<0.001	−0.018(−0.025, −0.012)
INR value	0.014	<0.001	0.680(0.334, 1.026)
*CYP4F2* rs2108622	0.007	0.010	0.163(0.039, 0.288)
Amiodarone use	0.006	0.013	−0.614(−1.099, −0.128)
Diabetes mellitus	0.006	0.018	0.370(0.063, 0.677)
Digoxin use	0.005	0.025	−0.252(−0.473, −0.032)

Total R^2^ for the model 45.1%. Coefficient B means the coefficient of the variables in multivariate linear regression model.

Warfarin maintenance dose (mg/day)  = 0.135+1.781×rs7294 - 1.214×rs1057910+1.288×BSA - 0.019×age+0.708×(target INR) +0.159×rs2108622+0.373×DM - 0.581×Amiodarone - 0.252×Digoxin

In total, the algorithm could explain 45.1% of the variability in warfarin dose.

### Efficacy of the algorithm for predicting the warfarin maintenance dose

We calculated the predicted dose of warfarin with the algorithm in the validation cohort (n = 236). The efficacy of the novel algorithm was assessed by Pearson coefficient analysis. A moderately strong correlation between predicted and actual warfarin dose was observed (Pearson r = 0.609, *P*-value<0.001).

Furthermore, we evaluated the accuracy of our algorithm in subgroups distributed according to the warfarin dose range in the whole cohort (Table 5). In the intermediate dose group, the accuracy of the dose prediction was much higher than in the other two groups. In the intermediate dose group (70.1% in the whole cohort), low dose group (13.1% in the whole cohort), and the high dose group (16.8% in the whole cohort), 66.8%, 19.4%, and 43.9% of the dose predictions fell within 20% of the actual dose (ideal dose), respectively. Most of the predictions (71.8%) were overestimated in the low dose group; however, most of the predictions (56.1%) were underestimated in high dose group.

**Table 5:** Percentage of patients in the whole cohort with an ideal, underestimated or overestimated dose of warfarin estimated with algorithms derived in Chinese.

Actual dose required	Number of patients	Underestimation	Ideal dose*	Overestimation
<2 mg/day (low dose)	103			
Northern algorithm†		8.7	19.4	71.8
Central algorithm		5.8	20.4	73.8
Southern algorithm		0	23.3	76.7
2–4 mg/day (intermediate dose)	552			
Northern algorithm		13.6	66.8	19.6
Central algorithm		18.8	59.6	21.6
Southern algorithm		19.4	70.7	10.0
>4 mg/day (high dose)	132			
Northern algorithm		56.1	43.9	0
Central algorithm		67.4	31.8	0.8
Southern algorithm		83.3	16.7	0
Total	787			
Northern algorithm		20.1	56.8	23.1
Central algorithm		25.3	49.8	24.9
Southern algorithm		27.6	55.4	17.0

*The ideal dose was defined as a predicted dose that was within 20% of the actual stable maintenance warfarin dose, underestimation was defined as a predicted dose that was at least 20% lower than the actual dose, and overestimation was defined as a predicted dose that was at least 20% higher than the actual dose.

† Northern algorithm means the algorithm derived in a Chinese Han population who live in Northern China in our study; Central algorithm means the algorithm derived in a Chinese Han population who live in Central China in the Tan et al study [33]; and southern algorithm means the algorithm derived in a Chinese Han population lived in Southern China in the Zhong et al study [16].

### Comparing our algorithm with the two algorithms derived from southern and central Chinese populations

We further compared our algorithm with the other two algorithms derived from southern Chinese [16] and central Chinese [33] populations. The two algorithms are Square root of Warfarin maintenance dose (mg/day)  = 1.68143 – 0.0029*age +0.30784*BSA – 0.2633*(*VKORC1* g.3588G>A) – 0.19114*(CYP2C9*3) +0.14735*(*CYP4F2* c.1297G>A) – 0.1797*amiodarone – 0.4138*fluconazole – 0.1888*diltiazem and Square root of Warfarin maintenance dose (mg/day)  = 2.140-0.370*(*VKORC1*-1639 G>A) – 0.332* (CYP2C9*3) +0.324 *BSA -0.004*age-0.231*(number of increasing INR drugs) +0.105*(smoking habit) -0.135*(preoperative stroke history) – 0.108* (hypertension), respectively.

Our algorithm could explain 45.1% variability of warfarin dose. This level of accountability compares favorably with southern algorithm and central algorithm, which were 43.6% and 56.4%, respectively. Table 5 shows the percentage of patients in the entire cohort with an ideal, underestimated or overestimated dose of warfarin estimated with the three algorithms. Our algorithm and the southern algorithm showed better performances for the intermediate-dose group, our algorithm and central algorithm showed better in high-dose group, whereas the other two algorithms showed improved performances in the low dose group. In the whole cohort, our algorithm was the most accurate following Pearson correlation analysis; the coefficients were 0.648, 0.573, and 0.569, for our algorithm, the southern algorithm, and the central algorithm, respectively. (All *P*-values <0.001)

## Discussion

Our study is the first to develop an algorithm for predicting warfarin maintenance from a relatively large population in the northern area of China. Our algorithm could explain the 45.1% variability of warfarin dose observed in northern Chinese individuals. Common genetic variants could explain the 35% variability of warfarin maintenance dose, and common non-genetic factors could explain 10% variability.

### Genetic factors contribute most of variability of warfarin dose

Compared with previous reports regarding the *VKORC1* haplotype and *CYP2C9* *3, we also observed that they made the most contributions to warfarin dose. Three variants, rs1057910 (*3), rs4917639, and rs9332127 in the *CYP2C9* gene have been reported to be associated with warfarin dose [12], [22], [24]. Among these variants, rs9332127 was not detected in Caucasian individuals but was reported to affect warfarin dose in Chinese people [22], [24], [35]; however, no significant association was observed in Indonesian populations [36]. In our study and previous studies [12], [37], univariate analyses showed that rs4917639 was significantly associated with warfarin dose. However, in stepwise multiple regression studies, only rs1057910 was retained in our final algorithm. In a study of Chinese individuals by Liu et al, no association of rs4917639 with warfarin was observed [37]. We speculated that rs1057910 noticeably affected warfarin dose and the association between rs4917639 or rs9332127 and warfarin dose was derived from the linkage disequilibrium. We calculated the D′ and r^2^ between rs1057910 with the other two variants, which were D′ = 1, r^2^ = 0.52 between rs1057910 and rs4917639, and D′ = 0.09, r^2^ = 0.01 between rs1057910 and rs9332127, respectively. However, the D′ and r^2^ were 0.97 and 0.44 between rs4917639 and rs9332127, respectively. Therefore, the association of rs4917639 or rs9332127 with warfarin dose is reflective of the moderate LD between the two SNPs and CYP2C9*3 rs1057910. In our study, we observed that *P*-values of the correlation between rs1057910, rs4917639, and rs9332127 and warfarin dose were 6.3×10^−8^, 1.5×10^−5^, and 0.374 in univariate analyses, respectively.

The enzyme encoded by the *CYP4F2* gene catalyzes the conversion of vitamin K to hydroxyl vitamin K and acts as a counterpart to VKORC1 in limiting the accumulation of vitamin K in hepatocytes [38]. A genome-wide association in Europeans revealed an association of the warfarin dose requirement with rs2108622 in *CYP4F2*, but only after adjusting for *CYP2C9* and *VKORC1* [13]. In the present study, the *P*-value of the correlation was 0.152 in univariate analysis, however, the *P*-value was 0.01 in the final multiple regression model. We further evaluated the effect of rs2108622 on warfarin dose in patients grouped by *VKORC1* rs7294 due to the significant effect of the *VKORC1* haplotype on warfarin dose and confirmed that the association between *CYP4F2* rs2108622 might be masked by *VKORC1* variants.

CYP4F2 is clearly involved in warfarin metabolism. In addition to rs2108622 Val/Met, the rs3093105 Trp/Gly is a non-synonymous substitution and might be functional [26] but we did not observe its association with warfarin dose. In related genes such as *GGCX*, *CALU*, *CYP2C19*, *EPHX1*, *F2*, *F5*, *F11*, and *PROCR*, we could not confirm the previous associations of these SNPs with warfarin dose in our Chinese population.

### Some non-genetic factors should be included in the algorithm

The non-genetic factors, age, BSA and target INR value, contribute most to the variability of warfarin dose, which is consistent with previous studies [28], [29], [30], [31], [32], [33], [39], [40], [41], [42], [43]. In common concurrent medications and comorbidities, we found that DM status and treatment with amiodarone and digoxin should also be included in the algorithm. Usually amiodarone use was often found in algorithms constructed in previous studies. Diabetes is known to be associated with hypercoagulable states [44], [45], [46], [47], [48], [49]. In three previous studies, diabetes was included in the final regression model, but this resulted in an inconsistent effect of diabetes on warfarin dose. The Hillman [50] and Lenzini groups [39] found that diabetes was negatively associated with warfarin dose, however, the Garcia group [51] observed that diabetes was positively associated with warfarin dose. Our results showed that patients with diabetes had a relatively higher warfarin dose (3.36±1.52 mg/day vs. 3.07±1.25 mg/day, *P* = 0.091). In the final regression model, diabetes showed a significant association (*P* = 0.018).

Digoxin, amiodarone and warfarin are known to interact with each other [52], and this interaction could increase the concentration of these drugs. However, digoxin has not been previously entered into the algorithm for predicting the warfarin maintenance dose. In our study, patients using digoxin had a significant lower warfarin maintenance dose than patients who were not using digoxin. The mean doses were 2.92 mg/day and 3.18 mg/day, respectively, and the *P*-value for ANOVA was 0.007. In final algorithm, digoxin use also showed a significant association with dose (*P* = 0.025). The contribution of amiodarone to warfarin dose is more than digoxin. If many patients had other drugs increasing INR in addition to digoxin in one study, the contribution of digoxin might be masked by amiodarone or other drugs in increasing INR. In our derivation cohort of 89 patients with digoxin use, only 5 patients used amiodarone at the same time. Thus, we could determine the contribution of digoxin to warfarin dose.

In many previous studies, gender was used in the final regression models [53], [54]. However, gender was removed from our model, although this factor was significantly associated with warfarin dose in univariate analysis. We found that gender was removed from the model when the BSA was included. Our results showed that the BSA was larger in men than in women (1.82±0.17 m^2^ vs. 1.59±0.14 m^2^, *P*<0.001). Although men had a higher warfarin dose than women (3.18±1.25 mg/day vs. 2.98±1.29 mg/day, *P* = 0.032) this observation might be due to the difference of BSA.

The major limitation in this study is that all subjects were recruited from a single center, and significant differences in genetic background and environmental factors were previously described in Northern China compared with Southern China and Central China. Therefore, the study sample may not be representative of the entire Chinese population. Our study population lived in Northern China, and 45.1% of the variation in warfarin maintenance dose could be accounted for by genetic variants in *VKORC1*, *CYP2C9*, and *CYP4F2*, and nongenetic factors including age, BSA, target INR, DM status, and use of amiodarone digoxin. Furthermore, our algorithm demonstrated the best predictor in northern Chinese people compared with the other two algorithms derived from southern Chinese and central Chinese populations. Thus, our results could be useful in the clinical practice for warfarin treatment of Chinese people who live in Northern China.
